# Two predominant molecular subtypes of spinal meningioma: thoracic NF2-mutant tumors strongly associated with female sex, and cervical AKT1-mutant tumors originating ventral to the spinal cord

**DOI:** 10.1007/s00401-022-02474-9

**Published:** 2022-08-09

**Authors:** Lingyang Hua, Majd Alkhatib, Dino Podlesek, Leila Günther, Thomas Pinzer, Matthias Meinhardt, Silke Zeugner, Sylvia Herold, Daniel P. Cahill, Priscilla K. Brastianos, Erik A. Williams, Victoria E. Clark, Ganesh M. Shankar, Hiroaki Wakimoto, Leihao Ren, Jiawei Chen, Ye Gong, Gabriele Schackert, Tareq A. Juratli

**Affiliations:** 1 Department of Neurosurgery, Huashan Hospital, Shanghai Medical College, Fudan University, Shanghai, China; 2 Department of Neurosurgery, Carl Gustav Carus University Hospital, TU Dresden, Dresden, Germany; 3 Department for Pathology, Carl Gustav Carus University Hospital, TU Dresden, Dresden, Germany; 4 Core Unit for Molecular Tumor Diagnostics (CMTD), National Center for Tumor Diseases Dresden (NCT/UCC), Dresden, Germany; 5 Department of Neurosurgery, Translational Neuro-Oncology Laboratory, Massachusetts General Hospital Cancer Center, Harvard Medical School, Boston, MA USA; 6 Department of Medicine, Massachusetts General Hospital Cancer Center, Harvard Medical School, Boston, MA USA

Spinal meningiomas (SM) comprise 5–10% of primary meningiomas and up to 30% of spinal intradural tumors. SMs are usually sporadic, but rarely, they can develop in association with genetic diseases like neurofibromatosis type 2 or schwannomatosis [2, 4, 6]. While the mutational landscape of intracranial meningiomas has been extensively studied [3, 5, 11, 14], our understanding of the molecular profile of SM remains incomplete. To date, genomic studies in SMs have been underpowered to make significant conclusions about the correlations between main genomic driver alterations and clinical features of these tumors. Here, we sought to assess the mutational profile of WHO grade 1 SM and to investigate the clinical characteristics that correlate with the genomic status.

Targeted next-generation sequencing was performed using assays covering frequently mutated genes in meningiomas as previously described [8] and online resource. Moreover, we correlated clinical and imaging data with the molecular tumor status.

Our study cohort consisted of 50 patients with newly diagnosed SM WHO grade 1 (Table 1). Thirty-eight patients were female and 12 were male (female:male ratio of 3.2:1). The median age at diagnosis was 66 years (range 28–84 years). Cohort patients were included if they had suspected sporadic meningioma based on lack of family history of neurofibromatosis type 2, schwannomatosis, and/or lack of other meningiomas or CNS tumors. The mean follow-up time of our SM cohort was 60 months (range 6–288 months).

**Table 1:** Comparison between patient’s demographics and tumor features of *AKT1*- and *NF2*-mutant spinal meningiomas

	All tumors (*n* = 50)	*AKT1*-mutant (*n* = 15)	*NF2*-mutant (*n* = 32)	*P* value
Median age (years)	66	71	65	**0.032**
Female sex	38	7/15 (46.6%)	30/32 (94%)	**0.0006**
Thoracic spine location	28	4/15 (26.6%)	24/32 (75%)	**0.0034**
Cervical spine location	21	11/15 (73.3%)	7/32 (21.8%)	**0.0012**
Ventral or ventro-lateral location to spinal cord	28	13/15 (87%)	14/32 (40.6%)	**0.010**
Dorsal or dorso-lateral location to spinal cord	22	2/15 (13.3%)	19/32 (59.3%)	**0.0043**
Meningothelial histology	24	14/15 (93.3%)	7/32 (21.8%)	**0.0001**
Tumor calcification	17	None	17/32 (53.2%)	**0.0002**

Bold indicates significance (*P* < 0.05)

Two predominant recurrent mutations were observed: *AKT1*^*E17K*^ mutations were detected in 15 (30%, 7 females and 8 males) and *NF2* mutations in 32 (64%, 30 females and 2 males) patients. Both mutations were mutually exclusive. In three cases (6%, one female and two males), no known driver mutation was found. All detected *AKT1*^*E17K*^ mutations were confirmed using Sanger sequencing, as described above. Meningiomas with an *AKT1 *^*E17K*^ mutation harbored additionally *ATRX* (*n* = 2), *ARID1A* (*n* = 2), *TRAF7* (*n* = 1) and *POLR2A* (*n* = 1). Co-mutations in *NF2*-mutant meningiomas included *SMARCB1* (*n* = 3) and *PTEN* (*n* = 1) mutations (Supplementary Tables 1 and 2, online resource).

Upon examination of the clinical features of patient harboring these two mutations, distinct cohorts emerged. The median age of patients with *AKT1*^*E17K*^ mutations (71 years old) was significantly higher when compared with patients with *NF2*-mutant meningiomas (65.5 years-old, *p* = 0.032). Notably, *NF2*-mutant meningiomas had a near-complete female predominance (n = 30/32, 94%) when compared to the balanced female–male incidence of *AKT1-*mutant tumors (*n* = 7/15, *p* = 0.0006, Table 1). A tumor location in the thoracic spine was significantly more common in *NF2*-mutant meningiomas (75%) than in their *AKT1*-mutant counterparts (26.6%) (*p* = 0.0034). In contrast, meningiomas harboring an *AKT1* mutation were predominantly located in the cervical spine (73.3%) and 87% of *AKT1-*mutant meningiomas (*n* = 13) (compared to 43.75% of *NF2*-mutant meningiomas, *n* = 14) arose ventrally or ventro-laterally to the spinal cord (*p* = 0.010). In contrast to *AKT1-*mutant meningiomas, a substantial proportion of *NF2*-mutant meningiomas developed in the dorsal or dorso-lateral location to the spinal cord (59.3%, *p* = 0.0043, Fig. 1 and Supplementary Fig. 1, online resource). Consistent with intracranial meningiomas, the histologic subtype of *NF2-*mutant meningiomas was variable (7 meningothelial, 16 psammomatous, 4 transitional, 5 fibrous), while all but 1 *AKT1*-mutant meningioma showed a meningothelial histology (93.3%, *p* = 0.0001). None of the *AKT1*-mutant meningiomas showed calcifications in the preoperative MRI or CT scan, whereas all calcified meningiomas (*n* = 17) harbored a *NF2* mutation (*p* = 0.0002). Several prior reports have discussed tumor calcification as a potential risk for permanent neurological deterioration due to difficult surgical removal [9, 12]. In our series, four patients (8%) experienced local tumor recurrence with three of these cases harboring a *NF2* mutation and with tumor calcifications.

**Fig. 1:**
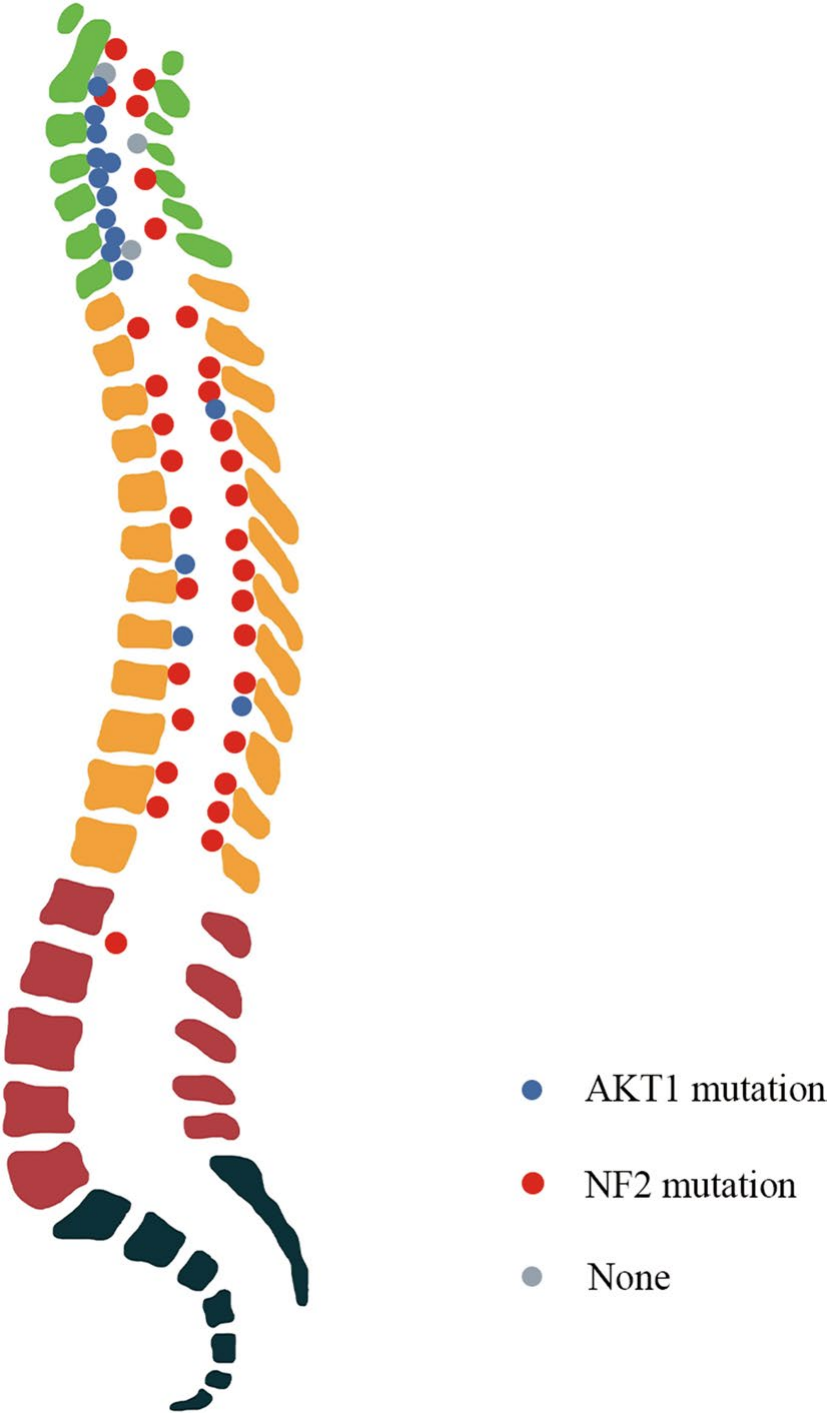
Anatomic distribution of *AKT1-* and *NF2*-mutant meningiomas along the spinal column and in relation to the spinal cord

Our data demonstrate the primary role of *NF2* and *AKT1* alterations as driver events in WHO grade 1 SM, as 94% of the cases harbored mutations in these genes. *AKT1* and *NF2* mutations presented in a mutually exclusive pattern, identifying unique clinical cohorts. In concordance with our findings, Arslantas et al. investigated 16 SM samples and described *NF2* mutations in 8 cases[1]. Remarkably, all *NF2*-mutant cases in the aforementioned study were located in the thoracic spine and six of them were female patients. Subsequently, Sahm et al. screened 1437 tumors for *AKT1* mutations and found 65 mutant cases, including 6 *AKT1*-mutant cases in 57 SMs [10], and described a strong association between *AKT1*^*E17K*^ mutations and spinal tumor localization.

In contrast to *AKT1*-mutant intracranial meningiomas, where *TRAF7* mutations are found to co-occur very frequently [5], we observed only a single *AKT1*-mutant SM case with a *TRAF7* co-mutation. Combining our data with these prior reports, focusing on WHO grade 1 meningiomas only, Clark et al. found *TRAF7* co-mutations in 50/68 *AKT1*-mutant intracranial meningiomas (15 were *AKT1* “isolated”, and 3 were co-mutant for *AKT1/NF2*), while we observed only 1 of 15 co-mutant tumors. Therefore, there appears to be a strong locational difference (*p* < 0.0001), suggesting that *AKT1*-mutant meningiomas arising from the ventral cervical spinal arachnoid are genetically distinct from their intracranial counterparts, based on the relative absence of *TRAF7* co-mutation. We speculate that the absence of *TRAF7* co-mutations in these tumors may potentially correlate with the more indolent clinical behavior that has been observed in *AKT1-*mutant SM compared to their intracranial counterparts.

Furthermore, we found two *SMARCB1* mutations that co-occurred in *NF2-*mutant meningiomas. *SMARCB1* mutations have been associated with the development of SM [2, 7]. Of note, our study included WHO grade 1 SMs exclusively and thus, did not include meningiomas with clear cell histology. This selection criterion may explain the absence of *SMARCE1* mutations in our study, which are known driver events in clear cell meningiomas [13]. Nevertheless, the presence of cryptic inactivation of *SMARCB1*, *NF2* or other genes cannot be definitely excluded in the three cases in our series that did not show known hot spot mutations in driver genes.

In summary, we have identified two predominant molecular subgroups in WHO grade 1 SM, characterized by *AKT1*^*E17K*^ and *NF2* mutations. Both mutations are mutually exclusive and are associated with distinct patient characteristics and tumor features. *AKT1*-mutant meningiomas originate in the cervical spine ventrally to the spinal cord, are almost exclusively associated with meningothelial histology and exhibit no calcifications on imaging. In contrast, *NF2*-mutant meningiomas show strong female gender predominance, arise with a wider anatomic distribution, although most frequently in the thoracic spine dorsally to the spinal cord, and can be calcified while displaying variable histologic subtypes (Supplementary Figs. 2 and 3, online resource).

## Supplementary Information

Below is the link to the electronic supplementary material.

Supplementary file1 (DOCX 907 KB)
