# Supplementary material for: Two predominant molecular subtypes of spinal meningioma: thoracic NF2-mutant tumors strongly associated with female sex, and cervical AKT1-mutant tumors originating ventral to the spinal cord
Source: Acta Neuropathol. 2022 Aug 9;144(5):1053–5. doi: 10.1007/s00401-022-02474-9 (PMC9547782; doi:10.1007/s00401-022-02474-9)
Supplement: Supplementary file 1 — Supplementary file1 (DOCX 907 KB) [file 401_2022_2474_MOESM1_ESM.docx]

**Two predominant molecular subtypes of spinal meningioma: thoracic NF2-mutant tumors strongly associated with female sex, and cervical AKT1-mutant tumors originating ventral to the spinal cord**

**Materials and Methods**

**Sample and data acquisition**

Patients with diagnosis of WHO grade 1 SM were retrospectively identified from two institutions databases. Samples were obtained from patients treated at Huashan Neurosurgical Center in China (n= 28) and at the University Hospital Dresden in Germany (n= 22). The study was approved by the human subject institutional review boards of Huashan Hospital, Fudan University (KY2020-1218) and the ethics committee at the University Hospital Dresden (BO-EK-1320320201). Written informed consent was obtained from all patients with samples undergoing sequencing. Cohort patients were included if they had suspected sporadic meningioma based on lack of family history of neurofibromatosis type 2, schwannomatosis, and/or lack of other meningiomas or CNS tumors. Moreover, radiation induced meningiomas were excluded from our cohort and none of the patients in the study had a history of a radiation therapy to the neck, chest or abdomen prior to developing a SM. Clinical data including age, sex, radiological features, tumor location, extent of tumor resection were retrospectively collected. The extent of resection was determined according to the Simpson grade system [4]. Simpson grade 1-2 was classified as gross total resection (GTR) and 3-5 as subtotal resection (STR). Follow-up was conducted at 6 months after surgery and annually thereafter.

Pathology was verified by the Departments of Pathology at Huashan and Dresden hospitals (HC and MM). All tumors were classified according to the 2016 WHO classification of tumors of the central nervous system.

**Neuroimaging**

Axial and sagittal T1-weighted post-contrast gadolinium-enhanced MR images as well as CT scans were collected in all cases. Radiological features comprising tumor location, tumor calcification, and ventral/dorsal locations in relation to the spinal cord were reviewed by two senior neurosurgeons (LH and TAJ).

**Tumor sequencing**

Due to the multi-institutional nature of the study, samples underwent targeted sequencing using two different Next Generation Sequencing (NGS) protocols.

NGS protocol at Huashan Neurosurgical Center (n= 28)

The Huashan Meningioma Panel, which was approved by CFDA in 2019, is a commercially used meningioma sequencing panel designed to detect all known mutations, copy number alterations and gene fusions in meningiomas [1, 2].

*DNA extraction and quantification*

The tumor DNA was extracted from 10 slides of 15 μm scrolls taken from archived formalin-fixed paraffin-embedded blocks. QIAamp DNA FFPE Tissue Kit (#56404) was used for DNA extraction of our FFPE samples, which were stored in the cold room (4℃). During the DNA extraction process, a repair solution mixed by different enzymes according to different proportions (Hieff NGS® Ultima DNA Library Prep Kit, Cat#12199) was used. Afterwards, DNA concentration was measured via NanoDrop spectrophotometer and the DNA quality was assessed using QIAseq DNA QuantiMIZE kits (QIAGEN), according to the manufacturer’s instructions. The extracted DNA was diluted to a concentration of 5‐10 ng/μL as a template, and PCR was performed using the QIAseq DNA QuantiMIZE kits.

*Design of the CNS tumor‐tailored 184‐gene NGS panel*

Amplicon based NGS sequencing (AmpliSeq) of a custom designed CNS panel (Illumina) was performed on a MiniSeq platform (Illumina, San Diego, USA), utilizing the Mid Output flow cell and sequencing cartridge. The self-designed CNS tumor panel contained 184 genes and four chromosomes as complete cds, which had been reported to be mutated in meningiomas. It includes the whole exomes of *NF2, TRAF7, KLF4, AKT1, SMO, PIK3CA, SMARCE1, BAP1, CDKN2A/B, ARIDIA, SUFU, SMARCB1, POLR2A, KDM5C, KDM6A, CHEK2, DMD, FAT1/2, KMT2D, PTEN, BRAF, PBRM1, STK11*, *IDH*, and *TERT*-promoter. In addition, CNV of four chromosomes, chr 22q, 1p, 14q and 10 are covered. All kinds of mutation types, including missense mutation, nonsense mutation, and frame-shift mutations were included in our analysis. The final NGS panel covers over 99.95% of the coding sequences of 184 genes.

*Library preparation and NGS*

Library synthesis was performed using the AmpliSeq Library Plus Kit (Illumina) according to the manufacturer's instructions and using the corresponding adaptor plates, containing in each well a premix of two index‐adaptors, each with an index length of 8 bp (Illumina). Libraries were pooled at a concentration of 10 nM. Runs were governed and bi‐directional sequencing with 2x 151 cycles was chosen with 2 × 8 bp index reads. The minimal sequencing depth of 300x was achieved for all genes (*TERT*-promoter not included).

NGS protocol at University Hospital Dresden (n= 22)

Fresh frozen tumor tissue was available from all 19 meningiomas. The tumor DNA was purified using AllPrep® DNA Universal Kit for fresh frozen tissue (Qiagen, Germantown MD) following the manufacturer’s instructions. The regions of interest were amplified using a custom designed amplicon panel according to the protocol “QIAseq Targeted DNA V3 Panel, May 2017” (QIAGEN, Hilden, Germany). The panel was custom-designed by our group to cover either mutation hotspots or—where loss of function is a known mechanism of action — whole coding exons [3]. The following meningioma-relevant genes were included: *AKT1, CDKN2A, KLF4, NF1, NF2, PIK3CA, PIK3R1, POLR2A, PTEN, SMARCB1, SMO, STAG2, SUFU, TP53, TRAF7*, and *TERT* promotor. During library preparation unique molecular barcodes and sample specific indices were incorporated according to the protocol. Indexed libraries were then quantified using a Qubit dsDNA HS Assay Kit (Thermo Fisher Scientific, MA, USA) and paired end sequenced (2x150 bp) on Illumina NextSeq platform. HG19 was used as reference genome for bioinformatic analyses.

For all 50 samples, further bio-informatics analysis was performed using the Biomedical Workbench from CLC (21.0.3) using a customized analysis algorithm with the following filters: coverage >/=100, allele frequency >/=5%.

**Sanger sequencing**

To validate the *AKT1^E17K^* mutations, Sanger sequencing was performed with primers as follows: Forward: CTGGCCCTAAGAAACAGCTCC Reverse: CGCCACAGAGAAGTTGTTGA. PCR amplification was carried out with a total volume of 10 μL containing 50 ng template DNA in Fast Start PCR Master Mix (Roche) following the manufacturer instructions.

**Statistical Analysis**

All analyses were performed with Stata 13.3 software (Stata Corp, College Station, TX). Continuous clinical features were described as the mean and standard deviations (SD). The Mann–Whitney U test and Fisher exact test were used to test for the association of clinical variables and NF2/AKT1 alterations. P values less than 0.05 were considered statistically significant.


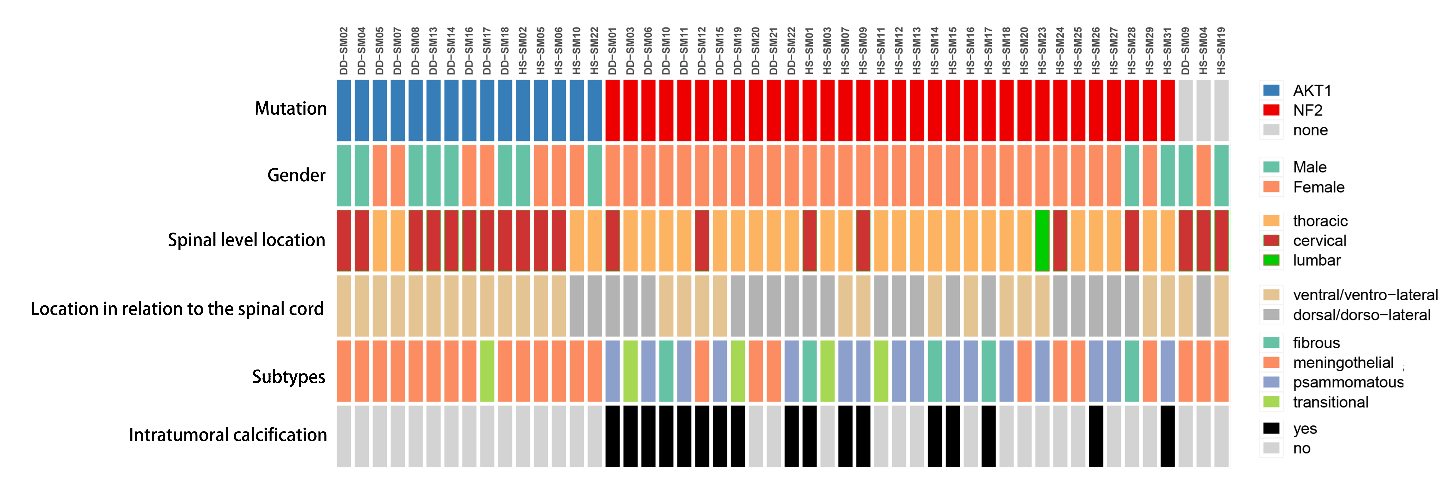


**Supplementary Figure 1**: Summary of clinical features and molecular alterations in *AKT1* and *NF2*-mutant spinal meningiomas.


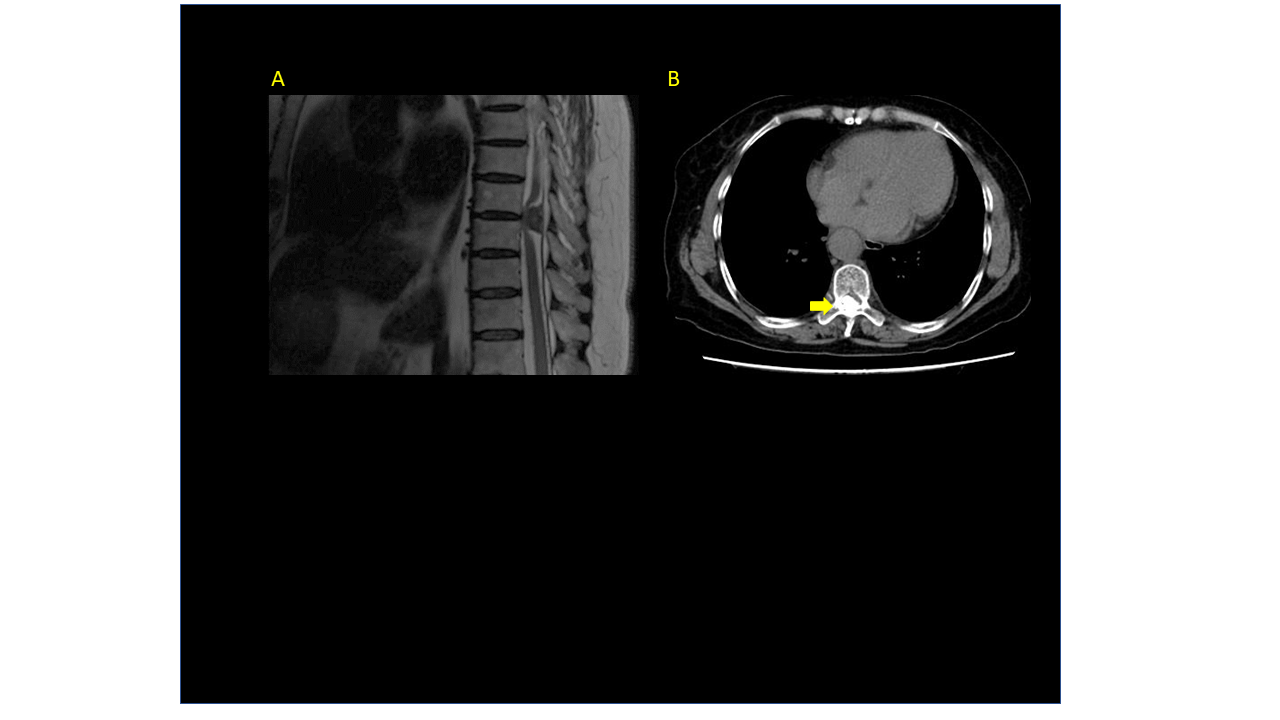


**Supplementary Figure 2:** A representative case of a 65-year-old female patient with a dorsally located T8-9 psammomatous spinal meningioma, with *NF2* mutation and significant intratumoral calcification. **A:** sagittal T2-weighted MRI. **B:** axial CT scan showing tumor calcification (yellow arrow).

**
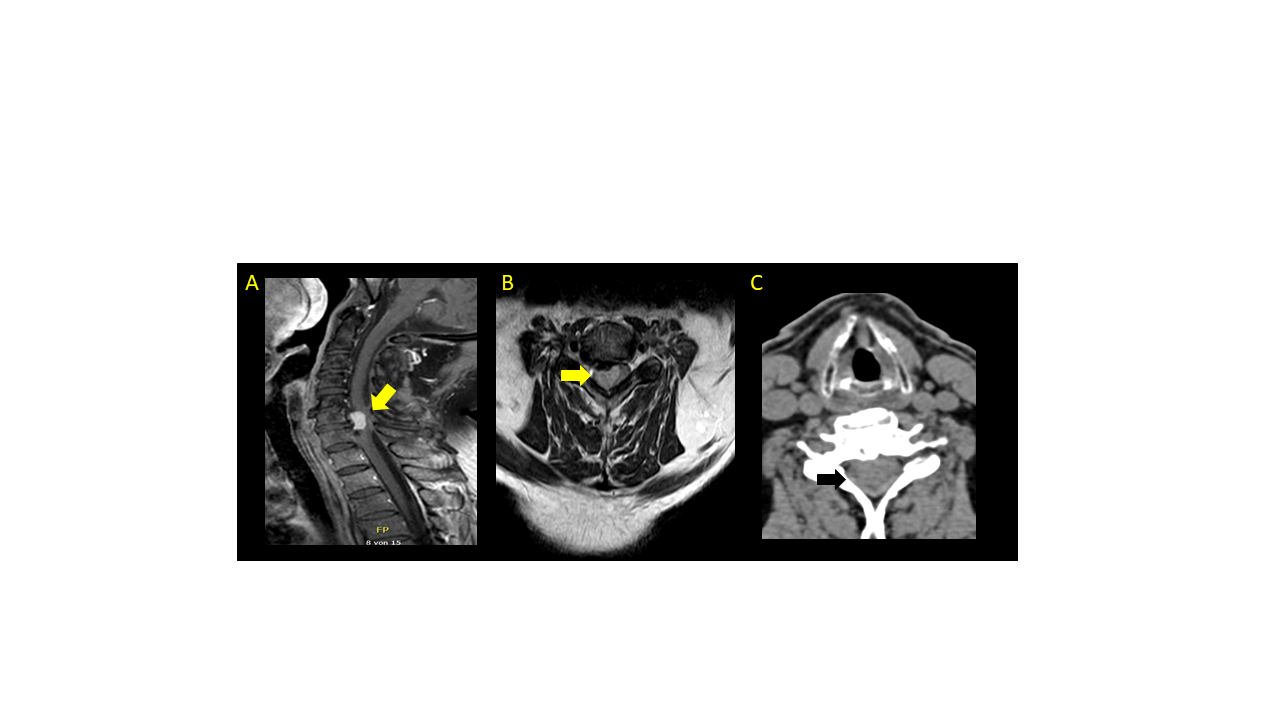
**

**Supplementary Figure 3:** An illustrative case of a 79-year-old male with a ventrally located, *AKT1*-mutant C6-7 meningothelial spinal meningioma, with no intratumoral calcification on the CT scan. **A:** sagittal T1-weighted MRI with gadolinium contrast (yellow arrow). **B:** axial T1-weighted MRI with gadolinium contrast (yellow arrow). **C**: CT scan showing no tumor calcification (black arrow).

**Supplementary Table 1: Patients characteristics and correlated tumor features.**

| **ID** | **Age (y)** | **Sex** | **WHO grade** | **Subtype** | **NF2 status** | **AKT1 status** | **Further mutant genes** | **Location in relation to the spinal cord** | **Spinal level location** | **Intratumoral calcification** |
| --- | --- | --- | --- | --- | --- | --- | --- | --- | --- | --- |
| DD-SM01 | 58 | F | 1 | psammomatous | mutant | WT |  | dorsal/dorso-lateral | cervical | yes |
| DD-SM02 | 73 | M | 1 | meningothelial | WT | mutant |  | ventral/ventro-lateral | cervical | no |
| DD-SM03 | 77 | F | 1 | transitional | mutant | WT | - | dorsal/dorso-lateral | thoracic | yes |
| DD-SM04 | 79 | M | 1 | meningothelial | WT | mutant | - | ventral/ventro-lateral | cervical | no |
| DD-SM05 | 66 | F | 1 | meningothelial | WT | mutant | ATRX | ventral/ventro-lateral | thoracic | no |
| DD-SM06 | 71 | F | 1 | psammomatous | mutant | WT | SMARCB1 | dorsal/dorso-lateral | thoracic | yes |
| DD-SM07 | 68 | F | 1 | meningothelial | WT | mutant | ATRX | ventral/ventro-lateral | thoracic | no |
| DD-SM08 | 58 | M | 1 | meningothelial | WT | mutant | PIK3R1 | ventral/ventro-lateral | cervical | no |
| DD-SM09 | 71 | M | 1 | meningothelial | WT | WT | - | ventral/ventro-lateral | cervical | no |
| DD-SM10 | 61 | F | 1 | fibrous | mutant | WT | - | ventral/ventro-lateral | thoracic | yes |
| DD-SM11 | 77 | F | 1 | psammomatous | mutant | WT | - | ventral/ventro-lateral | thoracic | yes |
| DD-SM12 | 63 | F | 1 | meningothelial | mutant | WT | - | ventral/ventro-lateral | cervical | yes |
| DD-SM13 | 45 | M | 1 | meningothelial | WT | mutant | SUFU | ventral/ventro-lateral | cervical | no |
| DD-SM14 | 52 | M | 1 | meningothelial | WT | mutant | - | ventral/ventro-lateral | cervical | no |
| DD-SM15 | 77 | F | 1 | psammomatous | mutant | WT | - | ventral/ventro-lateral | thoracic | yes |
| DD-SM16 | 78 | F | 1 | meningothelial | WT | mutant |  | ventral/ventro-lateral | cervical | no |
| DD-SM17 | 73 | F | 1 | transitional | WT | mutant | TRAF7 | ventral/ventro-lateral | cervical | no |
| DD-SM18 | 71 | M | 1 | meningothelial | WT | mutant | - | ventral/ventro-lateral | cervical | no |
| DD-SM19 | 66 | F | 1 | transitional | mutant | WT |  | dorsal/dorso-lateral | thoracic | yes |
| DD-SM20 | 80 | F | 1 | meningothelial | mutant | WT |  | dorsal/dorso-lateral | thoracic | no |
| DD-SM21 | 61 | F | 1 | meningothelial | mutant | WT | - | dorsal/dorso-lateral | thoracic | no |
| DD-SM22 | 84 | F | 1 | psammomatous | mutant | WT | - | dorsal/dorso-lateral | thoracic | yes |
| HS-SM01 | 60 | F | 1 | fibrous | mutant | WT | - | dorsal/dorso-lateral | cervical | yes |
| HS-SM02 | 57 | M | 1 | meningothelial | WT | mutant | - | ventral/ventro-lateral | cervical | no |
| HS-SM03 | 58 | F | 1 | transitional | mutant | WT | - | dorsal/dorso-lateral | thoracic | no |
| HS-SM04 | 35 | F | 1 | meningothelial | WT | WT | - | dorsal/dorso-lateral | cervical | no |
| HS-SM05 | 76 | F | 1 | meningothelial | WT | mutant | POLR2A | ventral/ventro-lateral | cervical | no |
| HS-SM06 | 80 | F | 1 | meningothelial | WT | mutant | ARID1A | ventral/ventro-lateral | cervical | no |
| HS-SM07 | 75 | F | 1 | psammomatous | mutant | WT | - | ventral/ventro-lateral | thoracic | yes |
| HS-SM09 | 60 | F | 1 | psammomatous | mutant | WT | - | ventral/ventro-lateral | cervical | yes |
| HS-SM10 | 73 | F | 1 | meningothelial | WT | mutant | ARID1A | dorsal/dorso-lateral | thoracic | no |
| HS-SM11 | 66 | F | 1 | transitional | mutant | WT | - | dorsal/dorso-lateral | thoracic | no |
| HS-SM12 | 57 | F | 1 | psammomatous | mutant | WT | PTEN | dorsal/dorso-lateral | thoracic | no |
| HS-SM13 | 65 | F | 1 | psammomatous | mutant | WT | - | dorsal/dorso-lateral | thoracic | no |
| HS-SM14 | 65 | F | 1 | fibrous | mutant | WT | - | ventral/ventro-lateral | thoracic | yes |
| HS-SM15 | 72 | F | 1 | psammomatous | mutant | WT | - | dorsal/dorso-lateral | thoracic | yes |
| HS-SM16 | 54 | F | 1 | psammomatous | mutant | WT | SMARCB1 | ventral/ventro-lateral | thoracic | no |
| HS-SM17 | 71 | F | 1 | fibrous | mutant | WT | - | dorsal/dorso-lateral | thoracic | yes |
| HS-SM18 | 68 | F | 1 | psammomatous | mutant | WT | - | ventral/ventro-lateral | thoracic | no |
| HS-SM19 | 52 | M | 1 | meningothelial | WT | WT | - | ventral/ventro-lateral | cervical | no |
| HS-SM20 | 38 | F | 1 | meningothelial | mutant | WT | SMARCB1 | ventral/ventro-lateral | thoracic | no |
| HS-SM22 | 56 | M | 1 | meningothelial | WT | mutant | - | dorsal/dorso-lateral | thoracic | no |
| HS-SM23 | 73 | F | 1 | psammomatous | mutant | WT | - | ventral/ventro-lateral | lumbar | no |
| HS-SM24 | 40 | F | 1 | meningothelial | mutant | WT | - | dorsal/dorso-lateral | cervical | no |
| HS-SM25 | 55 | F | 1 | meningothelial | mutant | WT | - | dorsal/dorso-lateral | thoracic | no |
| HS-SM26 | 77 | F | 1 | psammomatous | mutant | WT | - | dorsal/dorso-lateral | thoracic | yes |
| HS-SM27 | 57 | F | 1 | psammomatous | mutant | WT | - | dorsal/dorso-lateral | thoracic | no |
| HS-SM28 | 28 | M | 1 | fibrous | mutant | WT | - | dorsal/dorso-lateral | cervical | no |
| HS-SM29 | 68 | F | 1 | meningothelial | mutant | WT | - | ventral/ventro-lateral | thoracic | no |
| HS-SM31 | 67 | M | 1 | psammomatous | mutant | WT | - | ventral/ventro-lateral | thoracic | yes |

**Supplementary Table 2:** **Molecular data with detected single nucleotide variants**

| **ID** | **Mutation** | **Mutation frequency** | **HGVS** | **Gender** |
| --- | --- | --- | --- | --- |
| DD-SM01 | NF2 | 62.71% | c.431dupA(p.Tyr144fs) | Female |
| DD-SM02 | AKT1 | 28.66% | c.49G>A(p.Glu17Lys) | Male |
| DD-SM03 | NF2 | 53.32% | c.1423delA(p.Ile475fs) | Female |
| DD-SM04 | AKT1 | 47.67% | c.49G>A(p.Glu17Lys) | Male |
| DD-SM05 | AKT1 | 34.73% | c.49G>A(p.Glu17Lys) | Female |
| DD-SM06 | NF2 | 57.35% | c.834_837delGAAA(p.Lys279fs) | Female |
| DD-SM07 | AKT1 | 29.73% | c.49G>A(p.Glu17Lys) | Female |
| DD-SM08 | AKT1 | 33.39% | c.49G>A(p.Glu17Lys) | Male |
| DD-SM09 | none |  |  | Male |
| DD-SM10 | NF2 | 66.09% | c.955C>T(p.Gln319*) | Female |
| DD-SM11 | NF2 | 46.4% | c.1108delG(p.Ala370fs) | Female |
| DD-SM12 | NF2 | 57.27% | c.448-3_456delCAGTATGGTGAC(p.Gly151_Tyr153del) | Female |
| DD-SM13 | AKT1 | 33.07% | c.49G>A(p.Glu17Lys) | Male |
| DD-SM14 | AKT1 | 33.39% | c.49G>A(p.Glu17Lys) | Male |
| DD-SM15 | NF2 | 36.81% | c.1183_1184del(p.Ala395LysfsTer11) | Female |
| DD-SM16 | AKT1 | 24.32% | c.49G>A(p.Glu17Lys) | Female |
| DD-SM17 | AKT1 | 31.74% | c.49G>A(p.Glu17Lys) | Female |
| DD-SM18 | AKT1 | 58.3% | c.49G>A(p.Glu17Lys) | Male |
| DD-SM19 | NF2 | 51.50% | c.683delA(p.Lys228ArgfsTer23) | Female |
| DD-SM20 | NF2 | 66.83% | c.553delG(p.Glu185fs) | Female |
| DD-SM21 | NF2 | 65.12% | c.70del(p.Val24Ter) | Female |
| HS-SM01 | NF2 | 59.80% | c.860_861insTGAG(p.Ser288fs) | Female |
| HS-SM02 | AKT1 | 34.10% | c.49G>A(p.Glu17Lys) | Male |
| HS-SM03 | NF2 | 36.40% | c.1393G>T(p.Glu465*) | Female |
| HS-SM04 | none |  |  | Female |
| HS-SM05 | AKT1 | 10.70% | c.49G>A(p.Glu17Lys) | Female |
| HS-SM06 | AKT1 | 38.80% | c.49G>A(p.Glu17Lys) | Female |
| HS-SM07 | NF2 | 49.30% | NM_000268.3(NF2):c.169C>T(p.Arg57*) | Female |
| HS-SM09 | NF2 | 68.10% | NM_000268.3(NF2):c.351_353del(p.Leu117del) | Female |
| HS-SM10 | AKT1 | 40.60% | NM_001014431.1(AKT1):c.49G>A(p.Glu17Lys) | Female |
| HS-SM11 | NF2 | 49.00% | NM_000268.3(NF2):c.431dupA(p.Tyr144fs) | Female |
| HS-SM12 | NF2 | 13.60% | NM_000268.3(NF2):c.1458_1465del(p.Ile487fs) | Female |
| HS-SM13 | NF2 | 53.50% | NM_000268.3(NF2):c.663C>G(p.Tyr221*) | Female |
| HS-SM14 | NF2 | 55.10% | NM_000268.3(NF2):c.634C>T(p.Gln212*) | Female |
| HS-SM15 | NF2 | 35.10% | NM_000268.3(NF2):c.207_229del(p.Lys69fs) | Female |
| HS-SM16 | NF2 | 50.50% | NM_000268.3(NF2):c.126del(p.Lys44fs) | Female |
| HS-SM17 | NF2 | 24.30% | NM_000268.3(NF2):c.947del(p.Leu316fs) | Female |
| HS-SM18 | NF2 | 70.90% | NM_000268.3(NF2):c.822del(p.Lys274fs) | Female |
| HS-SM19 | none |  |  | Male |
| HS-SM20 | NF2 | 65.70% | NM_000268.3(NF2):c.600-3C>G | Female |
| HS-SM22 | AKT1 | 37.00% | NM_001014431.1(AKT1):c.49G>A(p.Glu17Lys) | Male |
| HS-SM23 | NF2 | 37.10% | NM_000268.3(NF2):c.809A>G(p.Glu270Gly) | Female |
| HS-SM24 | NF2 | 69.30% | NM_000268.3(NF2):c.551G>A(p.Trp184*) | Female |
| HS-SM25 | NF2 | 39.20% | NM_000268.3(NF2):c.1169_1193del(p.Ile390fs) | Female |
| HS-SM26 | NF2 | 3.10% | NM_000268.3(NF2):c.452G>A(p.Gly151Asp) | Female |
| HS-SM27 | NF2 | 50.60% | NM_000268.3(NF2):c.239del(p.Lys80fs) | Female |
| HS-SM28 | NF2 | 52.70% | NM_000268.3(NF2):c.1032del(p.Met345fs) | Male |
| HS-SM29 | NF2 | 60.00% | NM_000268.3(NF2):c.361C>T(p.Gln121*) | Female |
| HS-SM31 | NF2 | 31.90% | NM_000268.3(NF2):c.175_189del(p.Thr59_Gly63del) | Male |

**References**:

1 Deng J, Sun S, Chen J, Wang D, Cheng H, Chen H, Xie Q, Hua L, Gong Y (2021) TERT Alterations Predict Tumor Progression in De Novo High-Grade Meningiomas Following Adjuvant Radiotherapy. Front Oncol 11: 747592 Doi 10.3389/fonc.2021.747592

2 Jin L, Shi F, Chun Q, Chen H, Ma Y, Wu S, Hameed NUF, Mei C, Lu J, Zhang Jet al (2021) Artificial intelligence neuropathologist for glioma classification using deep learning on hematoxylin and eosin stained slide images and molecular markers. Neuro Oncol 23: 44-52 Doi 10.1093/neuonc/noaa163

3 Juratli TA, Prilop I, Saalfeld FC, Herold S, Meinhardt M, Wenzel C, Zeugner S, Aust DE, Barker FG, 2nd, Cahill DPet al (2021) Sporadic multiple meningiomas harbor distinct driver mutations. Acta Neuropathol Commun 9: 8 Doi 10.1186/s40478-020-01113-2

4 Simpson D (1957) The recurrence of intracranial meningiomas after surgical treatment. J Neurol Neurosurg Psychiatry 20: 22-39 Doi 10.1136/jnnp.20.1.22
